# Serum cytokines MCP-1 and GCS-F as potential biomarkers in pediatric inflammatory bowel disease

**DOI:** 10.1371/journal.pone.0288147

**Published:** 2023-11-03

**Authors:** Andrea Ott, Erol Tutdibi, Sybelle Goedicke-Fritz, Jakob Schöpe, Michael Zemlin, Nasenien Nourkami-Tutdibi

**Affiliations:** 1 Hospital for General Pediatrics and Neonatology, Saarland University Medical Center, Homburg/Saar, Germany; 2 Institute of Medical Biometry, Epidemiology and Medical Informatics, Saarland University, Homburg/Saar, Germany; Poznan University of Life Sciences: Uniwersytet Przyrodniczy w Poznaniu, POLAND

## Abstract

**Background:**

Inflammatory bowel diseases (IBDs) with the subtypes ulcerative colitis (UC) and Crohn disease (CD), are chronic autoimmune inflammatory disorders of the gastrointestinal tract. Cytokines are associated with the development and progression in pediatric IBD. We measured cytokine levels in pediatric IBD patients to assess their potential function as biomarkers in disease assessment.

**Method:**

In this prospective cohort study, we enrolled 33 children with IBD. All patients were in stable remission for 3 months on enrollment. Patients who developed a relapse within six months after enrollment were classified as relapsers. Blood sampling was performed at enrolment and for relapsers in relapse and post-relapse. Serum concentrations of 14 cytokines, chemokines and growth factors (IL-1α, IL-1β, IL-6, IL-12p40, IP-10, TNF-α, IFN-γ, IL-10, IL-8, MIP-1α, MCP-1, MCP-3, G-CSF, GM-CSF) were measured simultaneously using multiplex bead-based sandwich immunoassay on Luminex 100 system.

**Results:**

MCP-1 was significantly higher in CD patients compared to UC patients at each disease stage: stable remission (P<0.048), unstable remission (P<0.013), relapse (P<0.026) and post-relapse (P<0.024). G-CSF was significantly increased in UC patients developing a relapse and in post-relapse stage compared to UC patients in remission (P<0.02 and p<0.03, respectively).

**Conclusion:**

MCP-1 showed potential as a diagnostic biomarker in CD patients independent of disease activity as it was able to discriminate between subtypes of pediatric IBD. In UC patients, G-CSF was significantly elevated in relapsers indicating its use and role as a potential prognostic biomarker.

## Introduction

Inflammatory bowel disease (IBD) is a chronic disorder and characterized by chronic inflammation of the gastrointestinal tract with recurrent cycles of relapse and remission [1]. As per definition three major subgroups are differentiated: Crohn’s disease (CD), ulcerative colitis (UC), and IBD-unclassified (IBDU) [2–6]. A rising incidence, also within pediatric population is observed within the last decade [7–13]. While the pathology of pediatric IBD is not fully understood, IBD is an interplay of genetic and epigenetic factors accompanied by an imbalance of the intestinal microbiome [14]. Despite differences in clinical course of pediatric and adult patients, pathophysiology of the disease remains similar [15]. For adult IBD, 240 disease loci could be identified through genome-wide association studies most of them shared between CD and UC [16]. IBD variants affiliated with pediatric IBD have been analyzed with many of these variants being shared in adult IBD [17–20]. Next-generation sequencing enabled the possibility to diagnose refractory IBD and IBD-like diseases as rare monogenic disorders in comparison to polygenic disorders [21–24]. The incidence of pediatric IBD is 12-15/ 100,000 in industrialized countries like western Europe and North America [3, 25]. In Germany, Saxon Pediatric IBD Registry reported data, that the incidence of IBD in children and adolescents in Saxony increased at a similar rate as in other developed countries during the observation period [26, 27]. IBD is common in western countries, but there is a knowledge gap of epidemiologic data in newly industrialized countries. Reviews of adult-onset IBD have reported data from an increasing number of countries, where IBD is emerging and/or rapidly accelerating in incidence [28–34]. The overall global epidemiology of IBD in children and adolescents remains elusive. Timely diagnosis of pediatric IBD is important as diagnosis of IBD during childhood presents both psychological and somatic challenges for patients as missed school and linear growth delay [35–37]. Delay of diagnosis is still common in childhood IBD with a significant impact on clinical outcome [38–40].

IBD is accompanied with an interplay of the innate and adaptive immune system, genetic and epigenetic factors [41]. Gastrointestinal endoscopy has remained a reference standard but invasive test for the diagnosis, management, prognostics, and surveillance of IBD [42, 43]. Non-invasive approaches are favored in pediatric patients for follow-up investigations [44–47]. Single laboratory parameters are not sufficient to monitor patients with IBD. Here, multi-attribute measures of disease activity, as Pediatric Crohn’s Disease Activity Index (PCDAI) and Pediatric Ulcerative Colitis Activity Index (PUCAI) have been developed [48–53]. Classic inflammatory biomarkers like C-reactive protein (CRP), perinuclear anti-neutrophil cytoplasmatic antibodies (pANCA) and calprotectin are part of this assessment [54]. While the latter are used in clinical practice, different biomarkers in IBD have been evaluated and analyzed over the past two decades and are still part of clinical research. Since the last two decades, the suggestion of an existing network of regulatory cytokines that has important implications for disease progression in IBD becomes more evident [55, 56]. Cytokines, comprising interleukins, interferons, growth factors, chemokines, and tumor necrosis factor family, are soluble signaling molecules of the adaptive and innate immune system with an important role in the pathophysiology of IBD [57, 58]. Cytokines act on a local and systemic level, leading to the hypothesis that circulating cytokine levels may differentiate IBD from non-IBD patients, in a either diagnostic and/or prognostic function [59–61]. Analyzation of new data on the background to what is known about circulating cytokines may contribute to identify pathways in the development of IBD. Treatment response and complications differ between pediatric and adult IBD patients emphasizing the importance of an accurate diagnosis [15]. The most serious potential complications are intestinal or extra-intestinal malignancy [62]. Adequate treatment and the ability to diminish chronic inflammation might help to decrease short and long-term complications related to pediatric IBD.

Intestinal homeostasis depends on complex interactions between the microbiota, the intestinal epithelium, and the host immune system. Diverse regulatory mechanisms cooperate to maintain intestinal homeostasis. Breakdown of such pathways comprising the cytokine networks by which they are regulated may lead to IBD [63]. An imbalance between pro- and anti-inflammatory cytokines negatively influences the resolution of inflammation leading to disease perpetuation with consecutive tissue destruction. There is evidence that a network of regulatory cytokines has important implications for disease progression [56, 64]. Expression of cytokines, chemokines, and growth factors can be modulated during treatment regimens through steroids and/or monoclonal antibodies, both key drugs from pediatric IBD. Analyzing and evaluating serum cytokine, chemokine, and growth factor levels of patients with pediatric IBD at different stages of disease is crucial for a deeper comprehension and will aid physicians diagnosing and monitoring IBD. Cytokines and chemokines are elevated in mucosal tissue of IBD patients [55]. Most of the cytokines are functioning as chemokines with the ability to induce cellular mobilization and vascular permeability. Chemokines were regarded as cytokines with chemotactic functions. Their biological role is not limited to the recruitment of inflammatory cells though, but also on angiogenesis, hematopoiesis, and activation of immunocompetent cells. Studied due to their role in inflammation, chemokines and their receptors are mainly navigating mononuclear cells throughout the body, producing an immune response, discovered in the pathogenesis of various diseases. Chemokine receptors are important drug targets within the high number of molecules regulating inflammation and immunity [65]. Human growth factors include numbers of signaling molecules leading to ligand-specific signal transduction. Their downstream signaling affects cellular processes involved in epithelial wound healing. Deficiencies in certain growth factors in UC and CD patients, support the impact of human growth factors on the pathophysiological processes in IBD [66]. Transforming growth factor-beta 1 (TGF-β1), epidermal growth factor (EGF), keratinocyte growth factor (KGF), placenta-derived growth factor (PLGF), vascular endothelial growth factor (VEGF), granulocyte-macrophage colony-stimulating factor (GM-CSF) and finally granulocyte-colony stimulating factor (G-CSF) do play crucial roles in the pathogenesis of IBD [67–69]. Studies regarding cytokine levels in pediatric IBD patients are still sparse and little is known about serum cytokines that could distinguish CD from UC in pediatric IBD patients before starting treatment [61].

We hypothesize that significant differences may exist between serum cytokine, chemokine and/or growth factor profiles of pediatric IBD patients. We analyzed serum concentrations of cytokines, chemokines, and growth factors in pediatric IBD patients with UC and CD. We hypothesize that cytokines, chemokines and/or growth factors can potentially cluster UC and CD patients and may additionally be able to differentiate patients with active disease (relapse) and those in remission. In addition, we aimed to correlate cytokine levels with established laboratory markers as CRP and fecal calprotectin as well as clinical markers like the PUCAI and PCDAI scores.

## Methods

### Study population and design

In this observational single-center cohort study, a total of 33 pediatric patients with IBD were consecutively recruited and followed up between June 2015 and October 2017 from our specialist IBD clinic at the Children’s University Hospital in Homburg/Saar, Germany. The diagnosis of IBD and differentiation into CD or UC was done according to the Paris classification and is the most widely used tool for clinical classification of pediatric IBD based on endoscopic and radiological findings [70–72]. Biopsies were performed via gastroduodenoscopy and ileocolonoscopy. At least two tissue samples were taken from each affected bowel segment.

Eligible patients aged 17 years or below at IBD diagnosis. We excluded those participants with ambiguous IBD diagnosis, history of autoimmune/inflammatory diseases, follow-up time <6 months, or active disease within 3 months before study entry. Therapy was based on the clinical guideline program in Germany called “Arbeitsgemeinschaft der Wissenschaftlichen Medizinischen Fachgesellschaften e.V”(AWMF) for CD and UC which is in accordance with the recommendation of “The European Society for Pediatric Gastroenterology Hepatology and Nutrition” (ESPGHAN) and “North American Society For Pediatric Gastroenterology, Hepatology & Nutrition” (NASPGHAN) [73–80]. All patients remained under therapy with biological and/or immunomodulating drugs throughout the study. Furthermore, the patients had to have been in clinical remission at least over the past 3 months before inclusion. Routine clinic-visits were performed every 3-months or earlier in case of (suspected) relapse.

The disease activity was evaluated at visits using PCDAI and PUCAI indexes [48, 52]. An inactive phase of CD was defined as a PCDAI score of ≤10 and active phase as >10 points with a change of at least 5 points within 2 weeks since previous visit. In children with UC, inactive disease was defined as <10 and active phase as a PUCAI score of ≥10 with a change of at least 5 points within 2 weeks since last visit. An inactive phase of CD was defined as a PCDAI score of ≤10 and active phase as >10 points. In children with UC, inactive disease was defined as <10 and active phase as a PUCAI score of ≥10. The minimum follow-up period spanned 6 months if patient remained in clinical remission (remission group). In case of a relapse within 6 months after inclusion, the patients were followed until first remission after relapse (relapsing group). Patient characteristics and laboratory data were obtained from medical records. The study was approved by the ethical committee of the Saarland (reference no. 222/15) and informed consent was taken from all participants and their parents before enrollment. The PCDAI and PUCAI indexes utilized for this trial are made available as S1 Data.

### Blood sampling and processing

**Fig 1:**
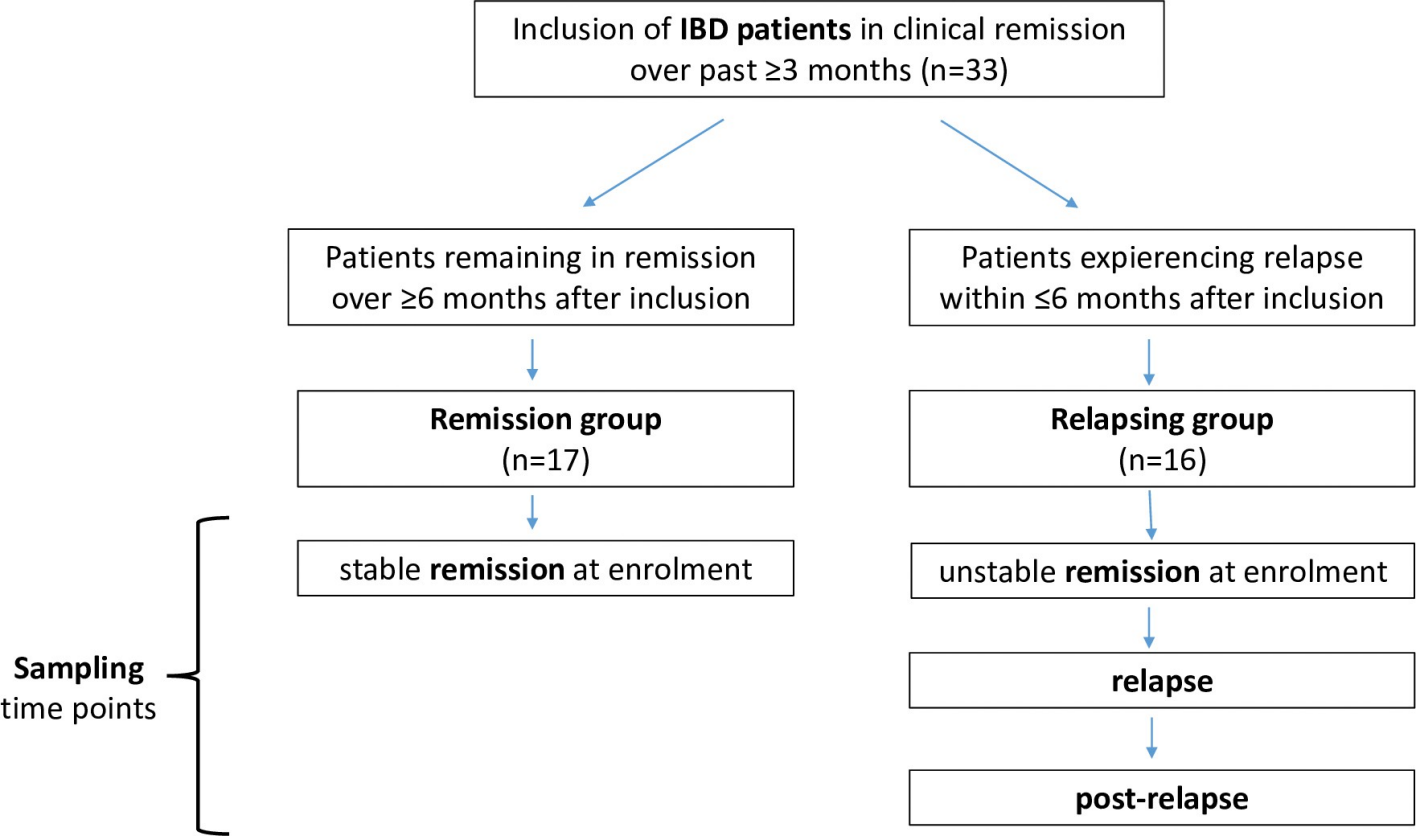
Study design with classification of IBD patients and samplings according to disease course after enrolment.

**Fig 2:**
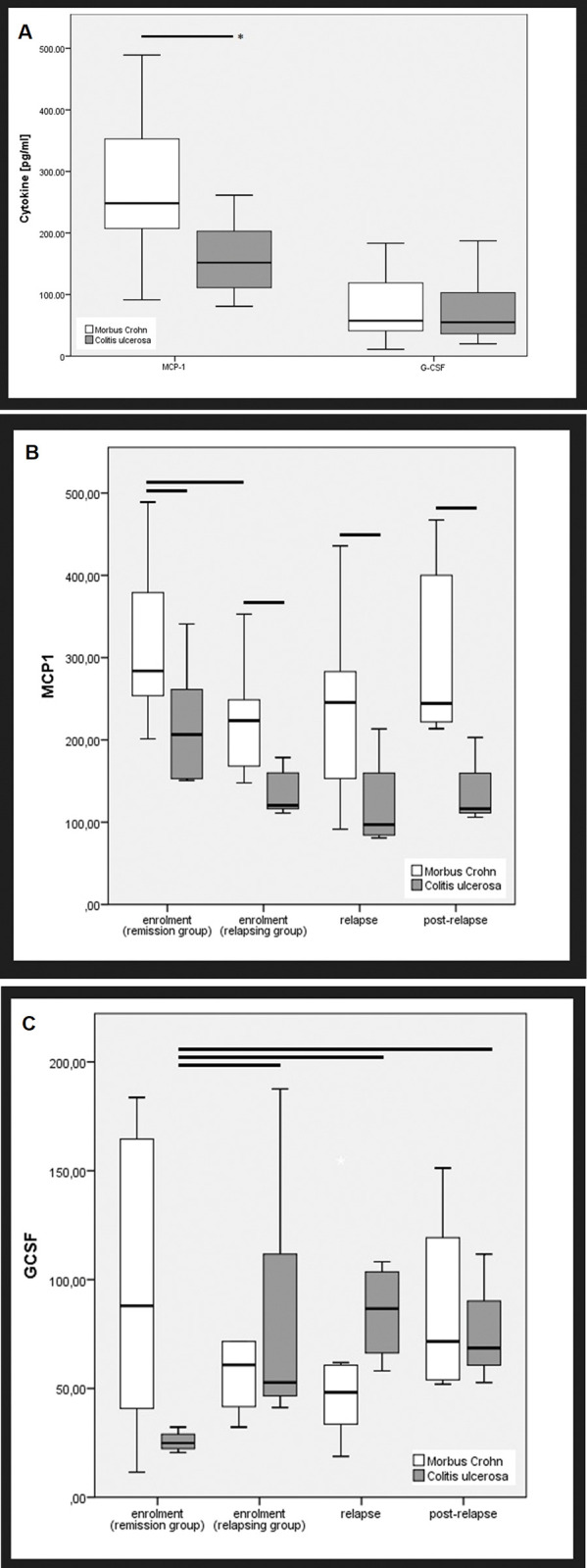
**a-c:** Serum concentrations (pg/ml) of MCP-1 and G-CSF in CD and UC patients. a) compares MCP-1 and G-CSF for CD versus UC, b) compares MCP-1 values for different time points in CD and UC patients and c) compares G-CSF levels for different time points in CD and UC. Data are presented as box plots by the method of Tukey. Comparison of cytokine values of CD with UC patients and of stable remission with relapsing group were performed using unpaired Mann-Whitney U-test. Cytokine concentrations within relapsing group (unstable remission to relapse and post-relapse) were analyzed by paired Wilcoxon test. *P<0.05 was considered as statistically significant.

Venous blood samples for routine laboratory tests and cytokine analysis were drawn during clinical visits. Within one hour after collection, the blood samples were centrifuged at 1500g at 4°C for 15 min, and then immediately the plasma was carefully separated from the blood clot and aliquoted into plain polypropylene tubes. Aliquots and remaining blood clots were coded and stored at -80°C until use. For the final study analysis, from each patient a baseline sample was available at enrolment and if they relapsed a second sample during active phase and a third sample was taken at recovery after relapse. Based on whether the patient remained in stable remission or later relapsed, the samples were divided into “stable or unstable remission” at enrolment, “relapse” and “post-relapse”, respectively (Fig 1 and Fig 2).

### Cytokine analysis

The simultaneous quantification of cytokine levels in plasma was performed as described before on a Luminex 100 system (Luminex Corporation, Austin, Texas, USA) with commercially available multiplex bead-based sandwich immunoassay. The Milliplex MAP human cytokine/chemokine panel (Cat# HCYTOMAG-60K, Millipore, Billerica, MA, USA) with a sensitivity range of 3.2–10,000 pg/ml was used to measure the concentrations of interleukin (IL)-1α, IL-1β, IL-6, IL-8, IL-10, IL-12p40, interferon gamma inducible protein-10 (IP-10), tumor necrosis factor α (TNF-α), interferon γ (IFN-γ), macrophage inflammatory protein-1α (MIP-1α), monocyte chemoattractant protein (MCP)-1, MCP-3, granulocyte-macrophage colony-stimulating factor (GM-CSF), and granulocyte colony-stimulating factor (G-CSF) according to the instructions provided by the manufacturer. All samples were run singly, and the results were quantified according to a standard curve. The MILLIPLEX Analyst software (version 5.1, Merck Millipore, Darmstadt, Germany), incorporating a 5-parameter logistic model for fitting the standard curve, was used to determine sample cytokine concentrations, expressed as pg/ml. Values of cytokine fluorescence intensities below background were set to missing. No further analysis was done on cytokines with non-detectable (missing) levels in more than 50% of samples. The lower limit of quantification (LLOQ) was set at the lowest standard concentration of 3.2 pg/ml for all analytes. For samples whose cytokine concentration was detected below the LLOQ, the value was set at LLOQ for the statistical analysis. All Luminex measurements were performed in the laboratory of the Children’s University Hospital in Homburg/Saar, Germany.

### Statistical analysis

The statistical analysis was performed using IBM SPSS Statistics version 26 software (Armonk, New York, United States). All results are presented as frequencies and percentages for categorical variables, and medians with minimum and maximum values for continuous variables. Categorical variables among groups were compared using Fisher’s exact test. Non-parametric Mann-Whitney U-test and the Kruskal-Wallis H-test were used for unpaired design. The Friedman test and the Wilcoxon test were carried out to analyze for variations within the relapsing group. Spearman’s correlation of rank coefficient was used to test correlation between measured cytokine values and clinical or laboratory markers of inflammatory. A value of P less than 0.05 was considered statistically significant. A post-hoc analysis was performed using G*Power version 3.1.3 to estimate the minimal detectable effect sizes with 80% power and 5% alpha. Our study was sensitive to detect large effect sizes of Cohen’s d ranging from 1.09 to 1.67 in the unpaired samples t-test and Cohen’s d from 0.96 to 1.76 in the paired samples t-test.

### Ethical statement

This study was approved by the Medical Ethical Committee of Saarland, Germany (reference no. 222/15) and was conducted in accordance with all ethical principles of the Seventh Revision of the Helsinki Declaration from 2013. Serum samples were obtained after patients, and their parents/guardians provided informed consent, and participation in the study was voluntary. All experiments were performed in accordance with the approved guidelines.

## Results

### Patient baseline demographic variables

Main clinical characteristics of the patients at enrolment are summarized in Table 1. The total IBD cohort consisted of 22 (66.7%) children with CD and 11 (33.3%) with UC, respectively. The IBD groups were comparable with respect to details of demographic and clinical data. Both median age at enrolment (16.4 years, range 8.2–19.1 years) and median duration of remission before study (19.3 months, range 3.5–108.1) showed no statistical difference between IBD groups. The median duration of disease differed significantly between IBD groups (CD: 31.3 months vs. UC: 9.2 months, P<0.02), while disease duration was comparable among the subgroups according to disease course. Seventeen patients stayed in remission (remission group) and 16 patients relapsed (relapsing group) during the follow-up period after inclusion. The rate of relapse showed no difference neither between CD and UC (50.0% vs. 45.5%, P<0.55) nor between male and female participants (47.8% vs. 50%, P<0.60). Type of localization, medication and main laboratory data did not differ significantly at enrolment between remission and relapsing groups. In contrast to CRP and calprotectin, clinical activity scores showed significant changes with course of disease. PCDAI and PUCAI scores were significantly higher during relapse compared to remission at enrolment and to post-relapse (Table 2).

**Table 1:** Characteristics of patients with Crohn’s disease (CD) and Ulcerative colitis (UC) at enrolment by remission and relapsing groups. Data are shown as frequencies and percentages for categorical variables, or median with minimum and maximum values for continuous variables. P were derived from Fisher’s exact test or Mann-Whitney U test. a) compared between the remission and relapsing group of CD patients. b) compared between the remission and relapsing group of UC patients. c) compared between CD and UC patients. P<0.05 was considered statistically significant. n.s. not significant.

	CD			UC			
	remission group	relapsing group	P a)	remission group	relapsing group	P b)	P c)
**Number (%)**	11 (50.0)	11 (50.0)	n.s.	6 (54.5)	5 (55.5)	n.s.	n.s.
**Male gender**	10 (90.9)	8 (72.7)	n.s.	2 (33.3)	3 (60.0)	n.s.	<0.049
**Age at diagnose, years**	11.2 (5.0–16.2)	11.5 (3.4–16.8)	n.s.	13.7 (12.2–15.7)	15.4 (11.7–16.5)	n.s.	<0.036
**Age at enrolment, years**	16.4 (9.6–19.1)	16.3 (8.2–18.4)	n.s.	15.0 (12.7–17.1)	17.1 (15.1–17.9)	n.s.	n.s.
**Disease duration, months**	35.2 (4.1–108.1)	23.2 (5.1–95.5)	n.s.	7.7 (3.5–47.9)	16.3 (3.5–65.7)	n.s.	<0.024
**Duration of remission before enrolment, months**	7.7 (3.7–60.3)	6.3 (3.6–90.5)	n.s.	5.3 (3.2–14.7)	5.4 (3.2–12.8)	n.s.	n.s.
**Localization, %**							
Ileal	18.2	36.4	n.s.				
Colonic	27.3	18.2					
Ileo-Colonic	45.5	9.1					
Upper gastrointestinal tract	9.0	36.3					
Ulcerative proctitis				33.3	0	n.s.	
Left-sided colitis				50.0	40.0		
Pancolitis				16.7	60.0		
**Medication, %**							
Corticosteroids	63.6	72.7	n.s.	100	100	-	n.s.
5-Aminosalicylic acids	18.2	9.1	n.s.	83.3	20.0	n.s.	<0.033
Azathioprine	81.8	81.8	n.s.	33.3	60.0	n.s.	<0.049
Methotrexate	0	9.1	n.s.	0	0	-	n.s.
TNF-alpha antagonist	18.2	27.3	n.s.	16.7	20.0	n.s.	n.s.
**Laboratory data**							
CRP, mg/dl	1.3 (0.3–32.4)	3.5 (0.3–20.4)	n.s.	1.2 (0.3–17.0)	0.9 (0.3–17.6)	n.s.	n.s.
Calprotectin, μg/g	19 (5–609)	107 (5–574)	n.s.	61 (5–2000)	51 (44–1750)	n.s.	n.s.
Hematocrit, %	40.9 (31.0–50.0)	39.0 (32.0–44.0)	n.s.	37.0 (28.0–45.0)	40.0 (36.0–44.0)	n.s.	n.s.
Albumin, g/dl	4.2 (3.4–4.5)	4.0 (3.4–4.2)	n.s.	4.4 (3.5–4.5)	4.5 (3.2–4.4)	n.s.	n.s.

**Table 2:** Disease activity markers of patients with Crohn’s disease (CD) and Ulcerative colitis (UC) according to disease stages. Data are shown as median with minimum and maximum values. P<0.05 was considered as statistically significant. a) significant difference between stable remission vs. relapse (Mann-Whitney U test). b) significant difference between stable remission vs. unstable remission (Mann-Whitney U test). c) significant difference between relapse vs. unstable remission (Wilcoxon test). d) significant difference between relapse vs. post-relapse (Wilcoxon test).

Markers	Disease stages	CD	UC
**PCDAI**	stable remission	5 (0–10)^a^	-
	unstable remission	7.5 (0–10)	-
** **	relapse	15 (12.5–50)^c,d^	-
	post-relapse	5 (0–10)	-
**PUCAI**	stable remission	**-**	0 (0–5)^a^
	unstable remission	-	5 (0–5)^b^
	relapse	-	40 (15–60)^c,d^
	post-relapse	-	5 (0–5)
**CRP, mg/dl**	stable remission	1.3 (0.3–32.4)	1.2 (0.3–17.0)
	unstable remission	3.5 (0.3–20.4)	0.9 (0.3–17.6)
	relapse	5.2 (0.3–20.2)	10.1 (4.9–45.6)
	post-relapse	3.6 (0.3–20.6)	0.7 (0.3–17.6)
**Calprotectin, μg/g**	stable remission	19 (5–609)	61 (5–2,000)
	unstable remission	107 (5–574)	51 (44–1,750)
	relapse	77 (13–2,000)	715 (667–2,000)
	post-relapse	5.2 (5–50)	86 (12–1,696)

### Cytokine levels compared to CD and UC groups

The detection rate of investigated cytokines and chemokines in the sampled sera of the patients varied between 82.8 and 100% (Table 3). IBD group, gender and disease activity was not related to detection rate. When we compared the cytokine levels between IBD groups, none of the interested markers exhibited a statistically significant difference except for the MCP-1 (P<0.006 Fig 2A). The concentration of the chemokine MCP-1 was significantly higher in CD patients compared to UC patients at each disease stage: stable remission (P<0.048), unstable remission (P<0.013), relapse (P<0.026) and post-relapse (P<0.024) as illustrated in Fig 2B. Other laboratory markers of inflammatory as fecal calprotectin and CRP did differ neither between IBD groups nor in subgroups according to disease stages.

**Table 3:** Serum cytokine levels (pg/ml) at enrolment of patients with Crohn’s disease (CD) and Ulcerative colitis (UC). Data are shown as median with minimum and maximum values. P<0.05 derived from Mann-Whitney U test was considered as statistically significant. n.s. not significant.

Cytokines	Detection rate	CD (n = 22)	UC (n = 11)	P
G-CSF	82.1%	63.9 (11.5–368.7)	41.3 (20.6–187.6)	n.s.
GM-CSF	100%	17.7 (3.2–141.2)	15.5 (9.8–65.4)	n.s.
IFN-γ	100%	17.5 (3.2–298.7)	32.2 (7.6–249.6)	n.s.
IL-10	94.6%	6.0 (3.2–43.1)	6.7 (3.2–45.2)	n.s.
IL-12p40	91.1%	31.7 (3.2–587.9)	42.4 (3.2–222.8)	n.s.
IL-1α	98.2%	7.8 (3.2–127.4)	3.2 (3.2–50.3)	n.s.
IL-1β	100%	3.2 (3.2–39.7)	3.2 (3.2–16.7)	n.s.
IL-6	92.9%	3.5 (3.2–33.2)	3.6 (3.2–13.3)	n.s.
IL-8	100%	6.0 (3.2–174.8)	4.6 (3.2–24.4)	n.s.
IP-10	100%	253.5 (67.4–1,231)	344.2 (82.1–779.2)	n.s.
MCP-1	100%	248.8 (147.7–763.8)	159.2 (111.1–340.9)	<0.006
MCP-3	100%	3.2 (3.2–1,697)	3.2 (3.2–191.3)	n.s.
MIP-1α	96.4%	15.9 (3.2–85.2)	9.8 (3.2–31.1)	n.s.
TNF-α	100%	8.2 (3.2–41.6)	8.1 (6.0–18.3)	n.s.

### Cytokine levels compared to remission and relapse

Within in the total IBD cohort, the levels of most cytokines at enrolment were comparable between remission and relapsing groups. However, the serum values of MCP-1 at enrolment were significantly higher in patients with stable remission than in those with unstable remission who experienced a relapse later (P<0.01).

When we restricted the analysis to each IBD groups, we found in CD, that levels of MCP-1 at enrolment were significantly higher in stable patients (remission group) compared to unstable patients developing a relapse during follow-up (relapsing group) (P<0.04, Fig 2B). Among UC values of G-CSF at enrolment were significantly increased in relapsing than in remission patients (P<0.02, Fig 2C). In addition, the comparison of cytokine concentrations between stable remission and relapse and post-relapse samples demonstrated again among UC patients, that G-CSF was significantly higher during relapse (P<0.03) and at post-relapse phase (P<0.03) compared to stable remission (Fig 2C). In contrast, we found such differences neither among CD group nor within the total IBD cohort.

Although stool calprotectin and CRP were elevated during relapse compared to those in stable remission, the difference was statistically significant only for CRP within the total IBD cohort (2.8 (0.3–20.4) vs. 1.3 (0.3–32.4) pg/ml, P<0.02). Values of cytokines, CRP and stool calprotectin remained unchanged in the relapsers for whom measurements were available before relapse at baseline, during relapse and after relapse. Neither cytokine concentrations nor inflammatory markers were significantly altered throughout the relapsing group in both CD and UC patients. Corresponding levels for relapse and post-relapse were comparable with remission.

Finally, we analyzed whether cytokine serum levels correlated with laboratory and clinical markers of disease activity. In total IBD cohort, there was a significant negative correlation between MCP-1 and calprotectin (r = -0.388, P<0.01), a positive association of TNF-a with CRP (r = 0.318, P<0.01) and of G-CSF with PUCAI (r = 0.465, P<0.04). Stool calprotectin was significantly correlated with CRP (r = 0.419, P<0.07) and with PUCAI (r = 0.492, P<0.04). In contrast, in CD and UC groups the cytokine levels were statistically independent of CRP, stool calprotectin and clinical activity markers.

## Discussion

### Biomarkers in IBD

Various biomarkers for pediatric and adult IBD have been investigated within the past decades. Biomarkers should be sensitive and disease specific. In small infants and children, biomarkers should be ideally non-invasive and/or easily accessible, preferably measurable by a waste product like stool or urine. Up to now, there is no ideal biomarker including all qualities for an accurate and timely diagnosis of IBD nor for differentiation of IBD subtypes. IBD biomarkers have been identified in colonic tissue, blood, stool, urine, and breath. Blood-based biomarkers can be easily obtained during routine diagnostics and are widely used. Much effort has been done by genome-wide association studies (GWAS) and multi-omic approaches to establish functional genomic network models identifying driver genes and providing a comprehensive characterization of disease architecture [18, 19, 81–87]. IBD-related genes have been demonstrated to organize into regulatory networks that significantly influence immune and inflammatory processes. There are also genetic variants and pathways implicated in pediatric IBD [18]. Cytokines play a crucial role in systemic and local inflammation, with chemotaxis and migration of immune cells to inflamed and injured tissues. They are key mediators of normal immune response and mediate cellular interactions in the intestine in both physiologic and pathophysiologic condition.

### Cytokines in IBD

Deregulation of cytokines and cytokine expression has a significant influence in disease pathogenesis.

Cytokines control and regulate multiple aspects of the inflammatory response. There is evidence that a network of regulatory cytokines has important implications for disease progression [56]. Studies in murine models demonstrated that cytokine function can be modulated for therapy purpose identifying cytokines as potential therapy targets [88, 89]. Novel therapeutic agents (e.g., anti-TNF-a, anti-IL-17, anti-IL-12/23, IL-1RA) that neutralize cytokines have been successfully translated into clinical practice [90, 91]. The incidence of pediatric IBD, comprising the subtyped CD, UC and IBDU is rising [25, 92–94]. There is evidence for cytokines and their impact on development and progression in IBD, yet data about pediatric IBD patients remains sparse [61]. Differences in blood sampling, analytic methods and data collection and analysis challenge researchers to gain sufficient and reliable data on cytokine patterns in pediatric IBD [95].

In the current study we measured several cytokines, chemokines, and growth factors with the aim to distinguish pediatric IBD subtypes from each other. We aimed to demonstrate that serum cytokine profiles can potentially cluster UC and CD patients and may additionally be able to differentiate patients with active disease and those in remission. Important functions and regulations of the measured panel according to pro- and anti-inflammatory functions of cytokines, chemokines, and growth factors will be discussed in the following section.

### Pro- and anti-inflammatory cytokines: IL-1α, IL-1β, IL-10, IL6, IL-8, IL-12p40, IFN-γ and TNF-α

Interleukin-1α, IL-1β and IL-10 are demonstrated to be key cytokines to control IL-23-producing monocytes [96]. IL-23 signaling and Th1/Th17 immunity are significant mediators of intestinal inflammation. Genetic mutations in IL-10-receptor are reported to result into very early onset IBD (VEO-IBD), with an onset before the age of 2 years [97]. IL-10 is an anti-inflammatory cytokine able to impede antigen presentation and pro-inflammatory cytokine release and therefore, regarded as the most important cytokine suppressing pro-inflammatory responses in the immune system. IL6, a pro-inflammatory cytokine influences intestinal inflammatory process. Children with newly diagnosed pediatric IBD, present with an enhanced and graded production of IL -6 in lamina propria of mucosal cells with serum IL-6 levels being positively correlated with active disease [98]. IL-6 is known to be associated with steroid resistance and disease activity in pediatric patients suffering from severe UC [99]. IL-8 is pro-inflammatory cytokine and important for the recruitment and activation of neutrophils and found to be elevated in newly and previously diagnosed pediatric IBD with higher concentrations. Additionally, IL-8 is being found in histologically more inflamed and damaged tissue segments in pediatric IBD patients [100]. IL-8 is not associated with clinical IBD subtype but part of both early and established mucosal inflammation in pediatric IBD [98]. IL-12p40 has been shown to suppress IFN-γ and is functioning as a biomarker in disease remission. A large study on pediatric IBD patients did not reveal any differences in circulating pro-inflammatory cytokine levels of UC and CD patients, but certain cytokines like IL-12p40, and TGF-β1 were found to be elevated in pediatric IBD patients in remission compared to those with active disease [68]. Recently one study reported that IL-7 may function as a potential biomarker to distinguish UC from CD in pediatric IBD patients [61]. Neutralizing TNF-α antibodies have been widely used to treat IBD in the clinical practice. Serum cytokine levels of TNF-α have been measured in many studies to assess efficacy and/or response to therapy with anti-TNF monoclonal antibodies [101–104].

### Chemokines: IP-10, MCP-1, MCP-3, MIP-1α

IP-10, a selective attractant of activated T-lymphocytes together with MCP-1 and MCP-3, chemokines that act on monocytes, activated T-lymphocytes, eosinophils, and basophils, are described as powerful players in the disease severity of patients with IBD. Systemic administration of MIP-1α significantly improved colitis in murine models [105].

### Growth factors: G-CSF and GM-CSF

The growth factors, GM-CSF and G-CSF are mainly responsible for macrophage and neutrophil trafficking. Neutralization of GM-CSF increases intestinal permeability and bacterial translocation and decreases neutrophil bacterial elimination. Similar has been described in the presence of elevated GM-CSF autoantibodies [106, 107]. GM-CSF autoantibodies can serve as a potential biomarker for confirmation of stable remission and prediction of relapses in patients with pediatric and adult IBD [105]. G-CSF has been described to be elevated in patients suffering from UC with recombinant G-CSF improving symptoms of IBD-like colitis in patients diagnosed with glycogen storage disease type Ib [108, 109].

In our study serum concentrations of cytokines, chemokines, and growth factors in pediatric UC and CD patients were measured and analyzed. We were able to demonstrate that some chemokines do have the ability to function as potential biomarkers helping to narrow the correct diagnosis and treatment. Single chemokines may have the potential to function as additional non-invasive markers to differentiate between disease entities and monitor disease activity. In the present study, we did not find decreases in pro-inflammatory cytokines with decreasing severity of the disease. Instead, we found that specific chemokines and growth factors to be statistically significant within our patient group. We will therefore discuss the potential as biomarkers on the analytes that showed to be statistically significant.

### Significant findings in scope of current research

#### Monocyte chemoattractant protein-1 (MCP-1/CCL2)

MCP-1 (chemokine nomenclature: C-C motif chemokine ligand 2 (CCL2)) is a member of the chemokine family, a group of small, secreted, chemotactic cytokines, named after their function of attracting cells. MCP-1 plays a critical role in innate immunity by directing the migration of monocytes into inflammatory sites. In IBD, specific chemotactic signals responsible for the attraction of peripheral blood monocytes recruited to the inflamed mucosa are not fully understood yet. Upregulation of various chemokines including MCP-1 in mucosal tissues could be demonstrated in clinical and experimental IBD [110, 111]. MCP-1 in combination with other chemokines are expressed in inflamed tissue and are induced in a variety of cell types in vitro through pro-inflammatory mediators like TNF-α, IL-1, and endotoxins [112, 113]. MCP-1 with its receptor CCR2 have central roles in monocyte recruitment during inflammation [66]. Isolation of MCP-1 created a revolutionary understanding of inflammation playing an important role also in cancer research, research that continues up to now. MCP-1 has become a molecular target to treat patients with many diseases [114, 115]. In patients suffering from COVID-19, several cytokines, and chemokines such as MCP-1 have been found to function as potential biomarkers [116, 117].

In our study, the concentration of MCP-1 was significantly higher in CD patients compared to UC patients at each disease stage: stable remission, unstable remission, relapse, and post-relapse phase. Furthermore, baseline MCP-1 was significantly altered in CD patients with stable remission than in those with unstable remission who later experienced a relapse. Chemokines and their receptors are also important in dendritic cell maturation, B- and T-cell development, T-helper cell type 1 (Th1) and Th2 responses, infections, angiogenesis, and tumor growth as well as metastasis. MCP-1 is a regulator of Th1 and Th2 effector function [118, 119]. Pathogenesis and inflammation in CD are associated Th1 response and Th17 cell response. Th17 cells mainly produce IL-17A. Research indicated that MCP-1 production is increased by IL-17A and IL-17A has been demonstrated to be increased in children with IBD [105]. Taken together MCP-1 might function as a sensitive biomarker in patients with CD as it is modulated through above-mentioned Th1/Th17 immune response possibly resulting in high levels of MCP-1 in CD independent of stage of disease. Studies suggest that IL-7 promotes a Th2 response with increased IL-10 and MCP-1, while decreasing Th1 cytokines, for example, IL-2, IFN-γ, and RANTES [120]. IL-7 was recently found to be highly elevated in pediatric CD patients and found to be a putative biomarker in pediatric IBD to distinguish CD from UC patients [61]. A significant difference could be described in the expression of IL-7 in serum from pediatric patients with CD [121]. Interestingly, IL-7 levels were significantly higher in patients in remission, compared with patients with active disease, which is in good accordance with our data showing that MCP-1 is higher in CD patients with stable remission in comparison to CD patients in relapse [68].

#### Granulocyte-colony stimulating factor (G-CSF/CSF3)

G-CSF is a cytokine with a crucial role in hematopoiesis of granulocytes and enhancement of neutrophil function. G-CSF does recruit progenitor and stems cells from the bone marrow into the peripheral blood. G-CSF has a major influence on different cell types, with anti-inflammatory effects on innate and adaptive immune cells [122, 123]. G-CSF treatment leads to an improvement of bowel inflammation, as demonstrated in animal colitis models and preclinical trials [124]. G-CSF plays a key role in maintaining immunological homeostasis in the intestine. Defects in the production of G-CSF may contribute to the pathophysiological process of IBD [125, 126]. G-CSF has been known to be elevated in patient with UC [60] and recombinant G-CSF has been proven to be a successful therapy in patients with CD [127]. Being gold standard in treatment of neutropenia, G-CSF has helped to achieve remission in pediatric patients with glycogen storage disease type Ib, who are predisposed to develop frequent infections and IBD-like colitis [128–130]. Murine models could demonstrate that G-CSF is upregulated in the microglia of colitis affected mice demonstrating its a crucial role in the pathogenesis of colitis [131].

In our study, G-CSF was significantly related to disease activity of UC patients. The values of G-CSF in the UC group were significantly higher in relapsing than in remission patients, making G-CSF a potential marker for disease activity in patients with UC. These findings are consistent with data from the literature as G-CSF is known to be elevated in patient with UC [60]. A hallmark of IBD is the migration of neutrophils to the colonic mucosa. Neutrophil accumulation in stool of patients suffering from UC positively correlates with active disease and infiltration of neutrophils is affiliated with disease severity [21, 132]. This might be an explanation of high G-CSF levels within UC relapsers and the crucial role of GCS-F in neutrophil trafficking.

### Strength and limitations

A major limitation of this study was the low number of patients from a single center. A higher number of patients might help to reveal differences among pediatric IBD subtypes with different immunopathogeneses. Another limitation of the study is that matched healthy controls are missing. In addition, pediatric IBD is a heterogenous disease, expression profiles of selected cytokines in biopsy specimens at different stages of disease are missing in our cohort. The major strength of our study is that we measured a wide range of different cytokines, chemokines, and growth factors in pediatric IBD patients at different time-points of disease activity. We were able to demonstrate that some cytokines like chemokines and growth factors might have the potential to serve as biomarkers in pediatric IBD.

## Conclusion

Easily accessible biomarkers for diagnosis and monitoring pediatric IBD are needed. Serological profiles of several cytokines allow for disease identification. We found, that serum levels of MCP-1 a regulator of T-cell commitment to Th1 and Th2 effector function, can function as a disease specific marker for CD irrespective of disease activity in CD patients. The serological profile of G-CSF in patients suffering from UC were significantly increased in relapsing than in remission phase making G-CSF putative biomarker to monitor disease activity marker in patients with UC.

## Supporting information

S1 Data Pediatric Crohn’s Disease Activity Index (PCDAI) and Pediatric Ulcerative Colitis Activity Index (PUCAI). (DOCX)

S2 Data STROBE statement—checklist of items that should be included in reports of cohort studies. (DOCX)

S3 Data (XLS)
