# Supplementary material for: Serum cytokines MCP-1 and GCS-F as potential biomarkers in pediatric inflammatory bowel disease
Source: PLoS One. 2023 Nov 3;18(11):e0288147. doi: 10.1371/journal.pone.0288147 (PMC10624322; doi:10.1371/journal.pone.0288147)
Supplement: Supplementary file 2 [file pone.0288147.s001.docx]

Paediatric Ulcerative Colitis Activity Index (PUCAI)	
**Item**	**Points**
**1. Abdominal pain**	
No pain	0
Pain can be ignored	5
Pain cannot be ignored	10
**2. Rectal bleeding**	
None	0
Small amount only, in <50% of stools	10
Small amount with most stools	20
Large amount (>50% of the stool content)	30
**3. Stool consistency of most stools**	
Formed	0
Partially formed	5
Completely unformed	10
**4. Number of stools per 24h**	
0–2	0
3–5	5
6–8	10
>8	15
**5. Nocturnal stools (any episode causing wakening)**	
No	0
Yes	10
**6. Activity level**	
No limitation of activity	0
Occasional limitation of activity	5
Severe restricted activity	10
**Sum of PUCAI (0–85)**	

Pediatric Crohn's Disease activity index (PCDAI)

**Item**	**Points**
**1. Clinical**	
Abdominal pain	
None	0
Mild	5
Moderate/Severe	10
Stool frequency	
One liquid stool, no bleeding	0
Up to 2 semi-formed with small blood, or 2-5 liquid	5
Gross bleeding, or ≥6 liquid, or nocturnal diarrhea	10
Patient activities and well-being	
No limitation of activities	0
Occasional difficulty maintaining activities	5
Frequent limitation of activities	10
**2. Laboratory**	
Haematokrit	
Normal	0
Mild decrease	2.5
Moderate/severe decrease	5
Erythrocyte sedimentation rate (mm/h)	
< 20	0
20-50	2.5
> 50	5
Albumin (g/dL)	
≥3.5	0
3.1-3.4	5
≤3.0	10
**3. Examination**	
Weight	
Weight gain or voluntary weight stable/loss	0
Involuntary weight stable, weight loss 1%-9%	5
Weight loss ≥10%	10
Height at diagnosis	
< 1 channel decrease	0
≥1, < 2 channel decrease	5
≥2 channel decrease	10
Height follow-up	
Height velocity ≥ - 1 SD	0
Height velocity < - 1 SD , > - 2 SD	5
Height velocity ≤ - 2 SD	10
Abdomen	
No tenderness, no mass	0
Tenderness, or mass without tenderness	5
Tenderness, involuntary guarding, definite mass	10
Perirectal disease	
None, asymptomatic tags	0
1-2 Indolent fistula, scant drainage, no tenderness	5
Active fistula, drainage, tenderness, or abscess	10
Extraintestinal manifestations (n)	
None	0
One	5
≥2	10
**Sum up PCDAI (0-110)**	
